# White Matter Hyperintensity Volume and Location: Associations With WM Microstructure, Brain Iron, and Cerebral Perfusion

**DOI:** 10.3389/fnagi.2021.617947

**Published:** 2021-07-05

**Authors:** Christopher E. Bauer, Valentinos Zachariou, Elayna Seago, Brian T. Gold

**Affiliations:** ^1^Department of Neuroscience, University of Kentucky, Lexington, KY, United States; ^2^Sanders-Brown Center on Aging, University of Kentucky, Lexington, KY, United States

**Keywords:** cerebral small vessel disease, DTI, white matter hyperintensities, cerebral perfusion, brain iron, QSM

## Abstract

Cerebral white matter hyperintensities (WMHs) represent macrostructural brain damage associated with various etiologies. However, the relative contributions of various etiologies to WMH volume, as assessed via different neuroimaging measures, is not well-understood. Here, we explored associations between three potential early markers of white matter hyperintensity volume. Specifically, the unique variance in total and regional WMH volumes accounted for by white matter microstructure, brain iron concentration and cerebral blood flow (CBF) was assessed. Regional volumes explored were periventricular and deep regions. Eighty healthy older adults (ages 60–86) were scanned at 3 Tesla MRI using fluid-attenuated inversion recovery, diffusion tensor imaging (DTI), multi-echo gradient-recalled echo and pseudo-continuous arterial spin labeling sequences. In a stepwise regression model, DTI-based radial diffusivity accounted for significant variance in total WMH volume (adjusted *R*^2^ change = 0.136). In contrast, iron concentration (adjusted *R*^2^ change = 0.043) and CBF (adjusted *R*^2^ change = 0.027) made more modest improvements to the variance accounted for in total WMH volume. However, there was an interaction between iron concentration and location on WMH volume such that iron concentration predicted deep (*p* = 0.034) but not periventricular (*p* = 0.414) WMH volume. Our results suggest that WM microstructure may be a better predictor of WMH volume than either brain iron or CBF but also draws attention to the possibility that some early WMH markers may be location-specific.

## Introduction

Cerebral white matter hyperintensities (WMHs) are diffuse regions of high signal intensity on T2-weighted magnetic resonance imaging scans (MRI; Hachinski et al., 1987; Wardlaw et al., 2015). The high signal intensity associated with WMHs reflect alterations in local tissue properties, including increased water content and myelin rarefaction (Fazekas et al., 1993; Pantoni and Garcia, 1997). WMHs are very common in older adults, with prevalence rates of ~60–80% in those above 65 years of age (de Leeuw et al., 2001; Wen and Sachdev, 2004). While non-specific in etiology, WMHs are generally associated with cerebral small vessel disease (cSVD; Pantoni and Garcia, 1995; Wardlaw et al., 2013a).

WMHs are thought to reflect “macrostructural” damage associated with moderate-to-advanced stages of cSVD (Debette and Markus, 2010; Gouw et al., 2011; Wardlaw et al., 2015). Consistent with this possibility, pathology studies suggest that WMHs are associated with structural brain changes such as gliosis, axonal degeneration, myelin loss and vacuolation (Gouw et al., 2011; Valdés Hernández et al., 2015). Further, a meta-analysis of 22 studies showed that WMHs were associated with progressive cognitive impairment, a 2-fold increase in the risk of dementia and a 3-fold increase in stroke risk (Debette and Markus, 2010). In addition, WMHs are associated with decreased physical ability (Sachdev et al., 2005), depression (Taylor et al., 2003) and increased risk of mortality (Debette and Markus, 2010).

A number of other MRI metrics have been suggested as potentially earlier markers of WMH volume (Wardlaw et al., 2013b; Smith and Beaudin, 2018). Identification of earlier neuroimaging markers of WMH volume is an important goal toward early identification of participants for enrollment in cSVD clinical trials (Charidimou et al., 2016; Boulouis et al., 2017). Ultimately, earlier markers of WMH will be defined as those which predict subsequent changes in vascular-related cognitive processes or WMH growth with high accuracy. Several studies have shown that baseline DTI-based measures in normal-appearing white matter (NAWM) predict WMH growth 1 to 2 years later (Maillard et al., 2014; Promjunyakul et al., 2018). However, while longitudinal studies are the gold-standard, they are also expensive and time-consuming. Results from cross-sectional studies may prove useful in guiding the selection of promising predictors of WMHs for use in future longitudinal studies. A number of previous cross-sectional studies have suggested significant relationships between either DTI metrics in NAWM (Maillard et al., 2011; Pelletier et al., 2016; Promjunyakul et al., 2016) or ASL (ten Dam et al., 2007; Brickman et al., 2009; Bahrani et al., 2017) and WMH volume. More recently, several studies have reported significant associations between brain iron and WMH volume (Yan et al., 2013; Valdés Hernández et al., 2016; Sun et al., 2017). However, no studies have considered these predictors simultaneously. Doing so is critical to identify which predictors account for the most unique variance in WMH volume after controlling for shared variance between predictors.

In addition to considering associations with total WMH volume, WMHs are often studied based on their specific location (Wardlaw et al., 2013a). In particular, specific regions of periventricular (PV) and deep WMH volume are often compared (Gouw et al., 2011; Griffanti et al., 2018). However, it remains unclear whether PV and deep WMHs reflect different pathologies (Gouw et al., 2011) or similar pathologies at different stages of cSVD severity (Ryu et al., 2014). Exploring potential interactions between predictors and WM location may help address this question.

Here, we explored the associations between several potential early stage markers of WMH volume. White matter (WM) microstructure, cerebral blood flow, and brain iron were used as predictors of WMH volume. Of key relevance, comprehensive models were used to assess the variance in WMH volume associated with each individual predictor, after controlling for variance associated with the other predictors to assess potential additive and synergistic effects on WMH volume and location. In addition, we explored whether each predictor contributed preferentially to PV or deep WMH volume.

## Materials and Methods

### Participants

Eighty healthy older adults (48 women, age range 60–86 years) were recruited for the experiment. All participants provided informed consent under a protocol approved by the Institutional Review Board of the University of Kentucky. Participants were recruited from an existing longitudinal cohort at the Sanders-Brown Center on Aging (SBCoA) and the Lexington community. Participants from the SBCoA were cognitively intact based on clinical consensus diagnosis and scores from the Uniform Data Set (UDS3) used by US ADRCs (Besser et al., 2018). Participants recruited from the community did not complete the UDS3 battery but were required to score 26 or more on the Montreal Cognitive Assessment (MoCA; Nasreddine et al., 2005) as a study inclusion criteria. Exclusion criteria were significant head injury (defined as loss of consciousness for more than 5 min), heart disease, stroke, neurological or psychiatric disorders, claustrophobia, pacemakers, the presence of metal fragments or any metal implants that are incompatible with MRI, diseases affecting the blood (anemia, kidney/heart disease etc.) or significant brain abnormalities detected during imaging. One participant was excluded from the sample due to the presence of an old stroke within the right motor cortex that was not clinically evident at study enrollment. Detailed characteristics of the final group of participants are shown in Table 1.

**Table 1:** Group demographics and Montreal Cognitive Assessment (MoCA^a^) scores.

	**Mean (S.D.)**	***N***
Age (Years)	70.4 (5.6)	80
Sex Ratio (F:M)	48:32	80
Education (Years)	16.4 (2.4)	80
MoCA^a^	27.1 (2.5)	69

*The table lists the mean (±sd) for age, the female/male ratio, and the mean (±sd) years of education and MoCA scores*.

a *Nasreddine et al., 2005*.

### Magnetic Resonance Imaging Protocol

Participants were screened to ensure magnetic safety for scanning within the Siemens Magnetom Prisma 3T (software version E11C) with a 64-channel head coil at the University of Kentucky's Magnetic Resonance Imaging and Spectroscopy Center (MRISC). The following scans were acquired: (1) a 3D multi-echo, T1-weighted Magnetization Prepared Rapid Gradient Echo (T1) scan, (2) a 3D fluid-attenuated inversion recovery (FLAIR) scan, (3) a diffusion tensor imaging (DTI) scan, (4) a 3D, multi-echo gradient-recalled echo scan used for quantitative susceptibility mapping (QSM), and (5) a pseudo-continuous arterial spin labeling (PCASL) perfusion scan. Several other sequences were collected during the scanning session related to other scientific questions and are not discussed further here.

The T1 sequence covered the entire brain [1 mm isotropic voxels, 256 × 256 × 176 mm acquisition matrix, repetition time (TR) = 2,530 millisecond (ms), inversion time = 1,100 ms, flip angel (FA) = 7°, scan duration = 5.88 min] and had four echoes [First echo time (TE1) =1.69 ms, echo spacing (ΔTE = 1.86 ms)]. The 3D FLAIR sequence covered the entire brain (1 mm isotropic voxels, 256 × 256 × 176 acquisition matrix, TR = 5,000 ms, TE = 388 ms, inversion time = 1,800 ms, scan duration = 6.45 min). The DTI sequence was acquired with 126 separate diffusion directions (2 mm isotropic voxels, 81 slices, TR = 3,400 ms, TE = 71 ms, scan duration = 7.45 min, posterior-to-anterior phase encoding direction) and 4 b-values (0 s/mm^2^, 500 s/mm^2^, 1,000 s/mm^2^ and 2,000 s/mm^2^). A short (28 s) reverse phase-encoding direction scan was also obtained with the same parameters as the main DTI scan and used to correct for susceptibility-induced distortions in the main DTI scan. The 3D spoiled gradient-recalled echo sequence was used for QSM (1.2 mm isotropic voxels, 144 slices, FA = 15°, 24 millisecond TR, scan duration = 6.3 min) with eight separate TEs (TE1 = 2.98 ms, ΔTE = 2.53 ms). A 3DGRASE PCASL sequence was used with 9 control-label pairs [3 segments (echo planar imaging factor = 63, turbo factor = 14), 9 tagged and 9 untagged volumes and 1 M0 for calibration, 3.4 × 3.4 × 4.0 mm voxels, 220 × 220 × 144 mm acquisition matrix (36 slices), TR = 5,070 ms, TE = 31.4 ms, FA = 120°, labeling duration = 2,025 ms, post-labeling delay = 2,500 ms, scan duration = 5.15 min].

### WMH Analyses

WMH volumes were computed using a validated 4-tissue segmentation method (DeCarli et al., 2013). Participants' FLAIR images were first registered to their own T1 [the four echoes averaged into a root mean square (RMS) image] image using FLIRT from FMRIB Software Library version 6.0.1 (FSL; Jenkinson et al., 2012), corrected for inhomogeneities using a previously published local histogram normalization (DeCarli et al., 1996), and then non-linearly aligned to a standard atlas (DeCarli et al., 2013). WMHs were estimated in standard space using Bayesian probability based on histogram fitting and prior probability maps. Voxels labeled as WMHs based on these maps also must have exceeded 3.5 SDs above the mean WM signal intensity. WMH volumes were calculated in participants' native FLAIR space after back-transformation and reported in cubic millimeters. WMHs were visually validated to ensure quality.

#### Periventricular and Deep WMH ROIs

**Figure 1:**
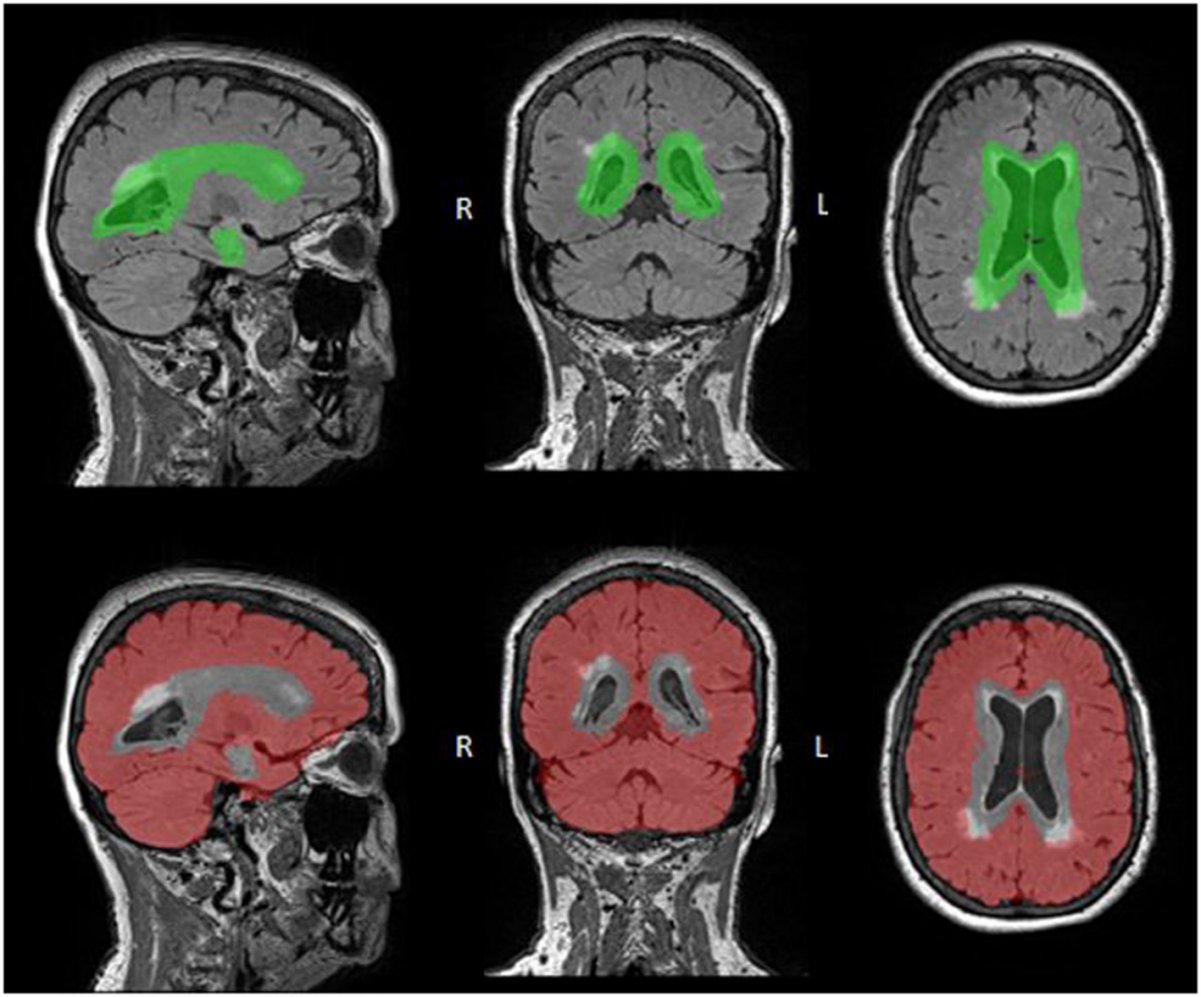
WMH Regional ROI Masks. PV (top: green) and deep (bottom: red) ROI masks are overlaid onto FLAIR images. These ROIs were defined by registering the ALVIN mask to each participant's native FLAIR space, with the area within the ALVIN mask defined as the PV ROI and the area outside defined as the deep ROI. Although both ROIs include all tissues, not just WMHs, these ROIs were multiplied by the participants WMH mask resulting in only WMH volume in each region.

WMH volume was further subdivided into periventricular (PV) and deep regions of interest (ROIs; Figure 1). PV WMHs were defined as being located within ~10 mm of the ventricles and deep WMHs were defined as those located outside this radius (Griffanti et al., 2018). These ROIs were delineated using a validated group mask of the lateral ventricles, the Automatic Lateral Ventricle delIneatioN (ALVIN) mask, which was created using data from 275 healthy adults (age range 18–94; Kempton et al., 2011).

In order to accomplish this, each participant's RMS T1 image was first skull-stripped and segmented using FreeSurfer 6.0 (Fischl et al., 2004). The resulting images were then aligned to the participants' FLAIR images using the analysis of functional neuroimages (AFNI) and a local Pearson correlation cost function. A binary mask was then created from these FLAIR aligned T1 images for later use in masking deep WMHs (described below). Subsequently, the aligned non-binarized T1 images were warped to MNI space using the MNI ICBM152 atlas (1 mm, 6th generation; Grabner et al., 2006) and a non-linear transformation. The inverse transformation matrix was then applied to the ALVIN mask (which is in MNI space) to bring it back to each participant's native space, using AFNI and a nearest neighbor interpolation method.

**Figure 2:**
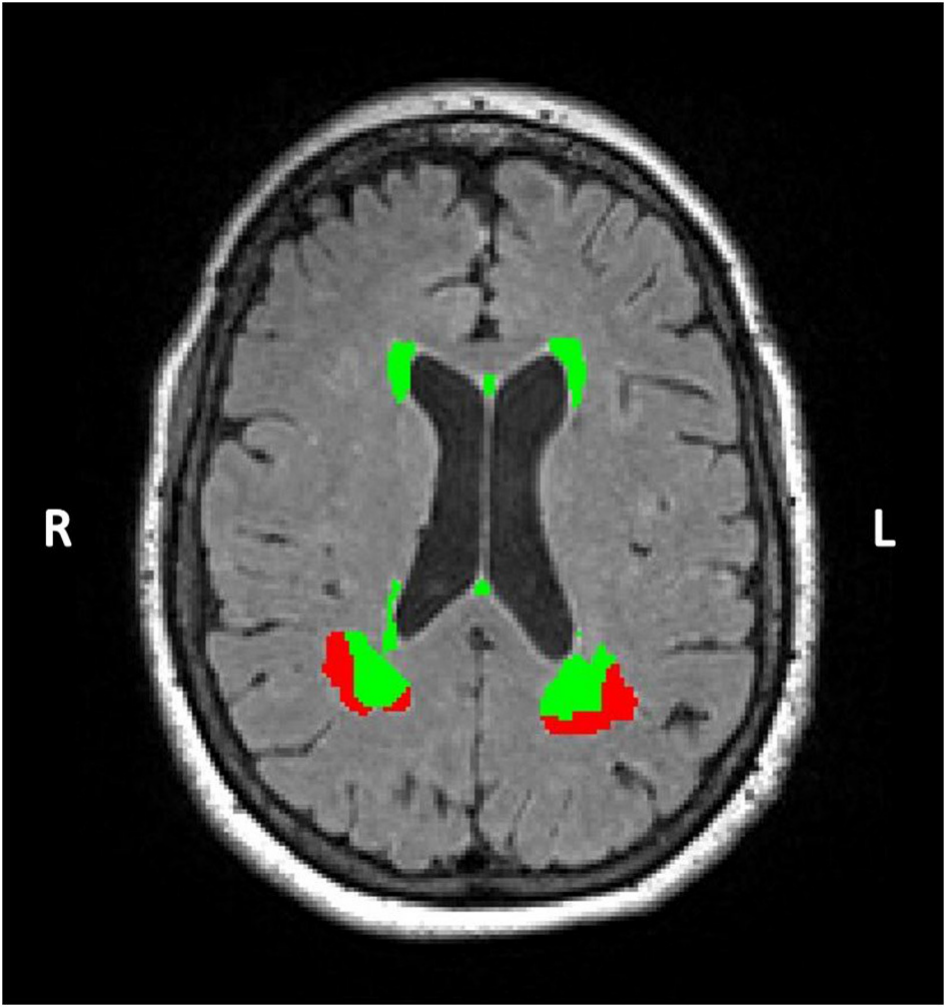
A Sample Segmented WMH Mask. In this axial FLAIR slice, WMHs in a representative participant are color-coded according to being in either PV (green) or deep (red) regions. WMH volume was extracted from each region separately for every participant.

The ALVIN mask extends beyond the lateral ventricles into brain parenchyma (~7–11 mm in most participants) to ensure inclusion of the lateral ventricles across participants with varying brain size. Typically, subsequent multiplication with a segmented CSF image is then performed to exclude parenchyma from the ventricular mask (Kempton et al., 2011). Here, rather than performing this step, each participant's WMH mask was multiplied by their native-space ALVIN mask (Figure 1, top panel, PV ROI) to produce PV WMH masks (Figure 2). Lastly, deep ROIs were created by subtracting the participant's native-space ALVIN masks from their binarized FLAIR aligned T1 masks (Figure 1, bottom panel). Each participant's deep ROI was then multiplied by their WMH mask to produce deep WMH masks (Figure 2).

### QSM Analyses

QSM images were processed in MATLAB using the Morphology Enabled Dipole Inversion toolbox (MEDI toolbox, release of 11/06/2017; Liu et al., 2012, 2013). This approach generates QSM images by inverting an estimate of the magnetic field to generate a distribution of local magnetic susceptibility used to calculate values for the underlying anatomy. The anatomical information required for MEDI was a (skull-stripped) magnitude image obtained during the same scan. The following steps were performed during MEDI: (1) non-linear fitting to the multi-echo data was used to estimate the magnetic field inhomogeneity. (2) Phase unwrapping using the magnitude image as a guide (Liu et al., 2013). (3) Removal of the background field by applying a projection onto the dipole field (Liu et al., 2011). (4) The remaining field was inverted to calculate the quantitative susceptibility map in parts per billion (PPB). (5) Local magnetic susceptibility in CSF was used to scale the QSM maps for susceptibility normalization such that positive values correspond to local magnetic susceptibility greater than CSF while negative values correspond to susceptibility less than CSF.

For the final step, ventricular reference masks were created for each participant by first registering the RMS T1 scans to the QSM magnitude image. The aligned RMS T1 scan was then used with ALVIN (Kempton et al., 2011) and SPM12 to create lateral ventricle masks for each participant, which were eroded by one voxel to prevent partial volume effects with surrounding gray and white matter. These masks were resampled to the QSM voxel resolution (1.2 mm isotropic) and used in the MEDI toolbox as the CSF reference mask (step 5).

#### Gray Matter QSM Measurement

Average GM QSM measures were calculated using FreeSurfer-derived ROIs from the cortex and dorsal striatum (caudate and putamen) as described in detail in our previous work (Zachariou et al., 2020). Briefly, these regions were selected based on previous literature indicating that iron in these areas increase with age (Aquino et al., 2009; Acosta-Cabronero et al., 2016). First, each participant's RMS T1 image was skull-stripped and segmented using FreeSurfer 6.0. Next, per participant cortical ROIs were created by combining the relevant GM segmented neocortical and allocortical (i.e., hippocampus) structures. Similarly, per participant subcortical ROIs were created from the FreeSurfer segmentations of the caudate and putamen.

The cortical and subcortical ROIs for each participant were then registered to their QSM images in native space using the following steps. First, each participant's RMS T1 was registered to the QSM magnitude image using the AFNI align_epi_anat.py function and a local Pearson correlation cost function. The resulting transformation matrices were then applied to each participants' cortical and subcortical ROIs using the AFNI function 3dAllineate with a nearest neighbor interpolation method. Finally, these ROIs were eroded by one voxel to prevent partial volume effects and were resampled to the QSM voxel resolution (1 to 1.2 mm isotropic voxels).

Due to significantly higher iron concentrations (over 10 times higher) in subcortical compared to cortical GM regions, per participant mean QSM values were extracted separately from the cortical and subcortical ROIs using fslstats. These values were then converted into z-scores and the mean of these z-scores across the 3 ROIs was used to create a single GM QSM measure for each participant.

### CBF Analyses

PCASL images were processed in FSL. First, all tagged/untagged pairs were motion corrected to the M_0_ image using FSL MCFLIRT. A perfusion image was subsequently calculated by taking the mean difference between the tagged and untagged pairs using asl_file in the BASIL toolbox (https://fsl.fmrib.ox.ac.uk/fsl/fslwiki/BASIL), which also helps correct for partial volume effects. Oxford_asl, which is also part of the BASIL toolbox, was then used for per-voxel calibration (using the M_0_ image) resulting in a calibrated map of tissue perfusion (ml/100 g/min).

#### Gray Matter CBF Measurement

Participants' FreeSurfer GM ROIs were registered to their PCASL images in native space using the following steps. Each participant's RMS T1 was aligned to their PCASL M_0_ image using AFNI and a local Pearson correlation cost function. The resulting transformation matrices were then applied to each participants' FreeSurfer GM ROIs using AFNI and a nearest neighbor interpolation method. Lastly, these ROIs were eroded by one voxel to prevent partial volume effects and were resampled to the PCASL voxel resolution (1 to 3.4 mm isotropic voxels).

Unlike with QSM, mean GM CBF signal was similar across the caudate, putamen, and cortical ROIs. Therefore, these ROIs were combined into a single ROI for our CBF measurement. Mean GM CBF signal was then extracted from the combined ROI using FSL.

### DTI Analyses

DTI was analyzed with FSL's tract-based spatial statistics (TBSS) pipeline (Smith et al., 2006), as described in detail in our previous work (Brown et al., 2016, 2019). Briefly, each participant's diffusion data was corrected for susceptibility induced field distortions using FSL's topup (Andersson et al., 2003), skull-stripped using BET (Smith, 2002), and non-linearly corrected for eddy currents and participant motion simultaneously with eddy (Andersson and Sotiropoulos, 2016). Greater than 2 mm subject motion per slice was used as a guideline for examination of excessive motion, although no slices from any participants were ultimately removed (minimal motion artifacts). The diffusion tensor model and eigenvalues (λ_1_, λ_2_, λ_3_) were computed within each voxel using DTIFIT, which were used to calculate fractional anisotropy (FA) images. After the initial preprocessing step (tbss_1_preproc), non-linear voxel-wise registration was used to transform each participant's FA image into MNI152 space, where they were averaged to create a mean FA image (tbss_2_reg and tbss_3_postreg). A common white matter tract skeleton was then created using the mean FA image. All participants FA data was then projected onto this skeleton which was thresholded at FA > 0.2 (tbss_4_prestats) to correct partial volume effects that may occur after warping across all participants. Radial diffusivity (RD) images were likewise processed with the same templates and transformations using tbss_non_FA.

#### DTI Masks of PV and Deep WM

The DTI ROI masks were generated from the JHU ICBM-81 WM ROI atlas (Mori et al., 2008). Several DTI ROIs were explored based on being major WM tracts with established connections between specific gray matter regions, located within either PV or deep WM regions and being well-aligned across participants in our DTI data. The PV ROI mask included the corpus callosum as it is the largest white matter structure in the brain connecting homologous structures across hemispheres (Fitsiori et al., 2011). Specific corpus callosum sub-sections were selected for being major partitions of the corpus callosum and well-aligned across participants (Splenium, Body, Tapetum). The deep ROI mask included the superior longitudinal fasciculus and external capsule which were chosen based on being major primary association fiber tracts in deep WM, and containing distant connections (connecting frontal and parietal regions and basal ganglia/claustrum with cortical regions, respectively; Mori et al., 2008). Furthermore, both tracts are believed to connect to cortical areas associated with several cognitive functions including language function, attention, and working memory/executive function (Mori et al., 2008; Wang et al., 2016; Nolze-Charron et al., 2020).

Mean FA and RD were extracted from each ROI tract, for each participant, using fslmeants. Mean values were computed across entire ROI WM tracts in order to provide anatomically meaningful diffusion estimates (i.e., estimates of tracts connecting specific gray matter regions). As a separate estimate, mean values were also computed across WM tracts after excluding values that overlapped with WMH voxels, resulting in ROIs restricted to only normal appearing white matter (NAWM). However, since the results were the same using each of these ROI methods, we present results using FA and RD values extracted from entire WM tracts to provide anatomically meaningful results. Extracted values from the entire ROIs were then used to create overall PV and deep FA and RD composite scores. These unweighted composite scores were created for each participant using the mean of the regional z-scored ROI tract values. This resulted in four composite scores for each participant: a PV FA composite score, a PV RD composite score, a deep FA composite score, and a deep RD composite score.

### Statistical Analyses

Statistical analyses were performed using SPSS (IBM, Chicago, IL, USA, version 26), with results considered statistically significant at p < 0.05. Data was considered a statistical outlier and removed if it was >3 standard deviations from the mean. All images for all participants were visually inspected for quality control. All predictors and dependent variables were tested for the assumption of normality using the Shapiro-Wilk test. Collinearity between predictors in all models was explored using the variance inflation factor (VIF), with a value of 5 implemented as a threshold value (Stine, 1995). CBF was z-scored across participants in the analyses to match the scale of the QSM and FA/DR composite scores. The coefficient of variation was calculated for unstandardized FA/DR, QSM, and CBF values in every ROI. This was done by dividing the standard deviation across participants by the mean signal across participants in every ROI. We also report the mean coefficient of variation across ROIs for FA/DR, QSM and CBF values. Finally, estimated total intracranial volume (eTIV) provided by FreeSurfer was z-scored across participants. This intracranial volume (ICV) measure was used as a covariate in subsequent analyses.

We first explored whether FA or DR better predicted PV and deep WMH volume (Section FA and RD as Predictors of WMH Volume). Separate linear regression models were run with FA and DR composite scores as the predictors and PV or deep WMH volume as the dependent variables. Age, sex, and ICV were included as covariates. The specific DTI metric, FA or RD, which had the larger effect size when predicting PV and deep WMH volume (partial r) was used in subsequent analyses.

Main effects of predictors on total WMH volume and predictor × WMH location interactions were initially explored using a general linear model repeated measures ANCOVA (Sections Imaging Modality Main Effects on WMH Volume and WMH Location Interactions and CBF Association With WMH Volume in Partial Models). CBF, QSM, and FA/DR, along with all possible interaction terms (CBF × QSM, QSM × FA/DR, and CBF × FA/DR) were the predictors of interest, with WMH volume as a 2-level dependent variable with PV and deep volume (not z-scored) as the levels. Age, sex, and ICV were included as covariates. For this analysis, white matter tracts from both PV and deep DTI ROIs were combined into a single FA/DR composite to reduce collinearity arising from including two DTI composites. We report the main effects of all predictors to examine effects on WMH volume more generally, and predictor interactions with WMH location to assess if any factors preferentially predict PV or deep WMH volume. Significant predictor by location interactions were followed-up with *post-hoc* analysis to identify relationships with WMH volume in each specific location (Section RD, CBF, and QSM Associations With Regional WMH Volumes). We were also interested in whether the accuracy of any of our predictors (CBF, QSM, or FA/DR) improves with increasing participant age. To test this possibility, we included age × predictor interaction terms in linear regression models predicting PV and deep WMH volume (Section Age Moderation Analysis).

Stepwise linear regression was used to further explore how much explained variance (*R*^2^) each predictor added regarding WMH volume (Section Additive Effects of WMH Predictors Using *R*^2^ Change). To accomplish this, we z-scored PV and deep WMH volume (the dependent variables used in the main ANCOVA model) across participants and then used the mean of the z-scores as our dependent variable (composite WMH volume). This was done to give equal weighting to WMH in both locations; using the more traditional total WMH volume would be largely driven by PV WMH volume which in general has much greater volume. The first step of the regression included only the covariates (age, sex, ICV). At each step, we added in the most significant predictor of WMH volume remaining as revealed by the initial ANCOVA. Interactions were excluded as they were not significant in the preceding analyses.

A retrospective power analysis was conducted to determine if our null between factor interaction findings were the result of insufficient power (Section Power Analysis). *Post-hoc* F-tests parameters were selected using G^*^power (version 3.1.9.2) with effect size, total sample size, number of tested predictors and total number of predictors specified. Cohen's f^2^ was used as a guideline for interpreting effect size.

## Results

### Participant and Data Characteristics

Participants summary demographic information is presented in Table 1. There were no outliers in any of the predictors (FA/DR composites, QSM composite, and CBF measurement) and one outlier in WMH data, which was excluded. Data from two other participants, one with a failed WMH segmentation and another with excessive motion in the QSM scan rendering it unusable, were also excluded. All the WMH distributions were skewed as is typical (PV; W statistic = 0.597; *p* < 0.001; Deep; W statistic = 0.318; *p* < 0.001) and were log transformed. The QSM composite and CBF measurement were normally distributed (*p* = 0.725; *p* = 0.669), while the FA/DR composites were mildly skewed but were not log transformed (W statistic = 0.924; *p* < 0.001), as this is not typically done in the DTI literature. Error residuals from all analyses were normally distributed indicating that the assumption of normality was met. Variance inflation factor for all predictors was <2 and tolerance was >0.5 in all analyses. The mean coefficient of variation was 0.0601 for FA (Superior Longitudinal Fasciculus ROI = 0.0585, External Capsule ROI = 0.0463, Body of Corpus Callosum ROI = 0.0556, Splenium of Corpus Callosum ROI = 0.0332, Tapetum of Corpus Callosum ROI = 0.107), and 0.0925 for DR (superior longitudinal fasciculus ROI = 0.0645, external capsule ROI = 0.0568, Body of Corpus Callosum ROI = 0.0955, Splenium of Corpus Callosum ROI = 0.0707, Tapetum of Corpus Callosum ROI = 0.175). The mean coefficient of variation was 0.362 for QSM (Caudate ROI = 0.395, Putamen ROI = 0.466, Cortical ROI = 0.225). The coefficient of variation was 0.174 for CBF (Gray Matter ROI = 0.174).

### FA and RD as Predictors of WMH Volume

Participant's DTI PV and deep composite scores (for FA and RD) were computed by z-scoring individual participant's mean values in each tract in the mask and then calculating the mean of those z-scores across tracts (Section DTI Masks of PV and Deep WM). RD had a larger effect size than FA in a model predicting PV WMH volume (RD partial *r* = 0.415, *p* < 0.001; FA partial *r* = −0.379, *p* = 0.001) and deep WMH volume (RD partial *r* = 0.323, *p* = 0.005; FA partial *r* = −0.141, *p* = 0.233). Therefore, RD was used as the DTI measure in PV and deep DTI ROIs in subsequent models.

### Imaging Modality Main Effects on WMH Volume and WMH Location Interactions

The ANCOVA indicated a main effect of DTI-based RD (*F* = 13.516, partial Eta = 0.412, *p* < 0.001) and QSM-based iron concentration (*F* = 5.107, partial Eta = 0.268, *p* = 0.027) on total WMH volume (Table 2). The main effect of CBF on total WMH volume was marginally significant (*F* = 3.530, partial Eta = −0.226, *p* = 0.065). There was a QSM × WMH location interaction [explored in *post-hoc* analysis below (Section RD, CBF, and QSM Associations with Regional WMH Volumes), *F* = 6.208, partial Eta = 0.293, *p* = 0.015] such that QSM positively predicted deep but not PV WMH volume, but no CBF × WMH location (*F* = 0.029, *p* = 0.866) or RD × WMH location interactions (*F* = 0.053, *p* = 0.819). There were no significant interactions between predictors.

**Table 2:** Summary of main effects and interactions of predictors on WMH volume and location.

**WMH Volume**	**Main Effects**		**Location Interaction**	
**Predictors**	**F-value**	***p*-value**	**F-value**	***p*-value**
Age	0.658	0.420	0.302	0.584
Sex	1.161	0.285	2.250	0.138
ICV	3.032	0.086	0.103	0.750
CBF	3.530	0.065	0.029	0.866
QSM	5.107	0.027^*^	6.208	0.015^*^
RD	13.516	<0.001^*^	0.053	0.819
CBF × QSM	0.573	0.452	0.689	0.410
QSM × RD	0.026	0.874	0.241	0.625
CBF × RD	0.018	0.894	0.127	0.723

*The F- and p-values for the main effects of predictors on total WMH volume are on the left, with the interaction effects of WMH location on the right. QSM and RD had robust main effects, while CBF had a marginally significant main effect on total WMH volume. Only QSM interacted with WMH location*.

* *p < 0.05*.

### CBF Association With WMH Volume in Partial Models

Our ANCOVA with all 3 contributing factors revealed that CBF was only marginally significant in predicting total WMH volume. However, several previous studies report associations between CBF and total WMH volume. Therefore, we further explored if CBF was significantly associated with total WMH volume in partial models of the ANCOVA with standard covariates age, sex, and ICV included. The purpose of this analysis was to determine which predictor may account for some of the variance in the CBF-WMH relationship.

With CBF as the only predictor of interest, CBF negatively predicted total WMH volume (*F* = 4.339, *p* = 0.041). CBF still predicted total WMH volume when QSM (and CBF × QSM interaction) was entered into the model but RD was omitted (*F* = 4.745, *p* = 0.033), yet was only marginally significant when RD was entered into the model but QSM was omitted (*F* = 3.777, *p* = 0.056).

### Additive Effects of WMH Predictors Using *R*^2^ Change

In step 1, covariates alone accounted for 20% of the variance in composite WMH volume (Adjusted *R*^2^ = 0.200; Table 3). Including RD in the model increased the variance explained to 33.6% (Adjusted *R*^2^ change = 0.136). Further additions of QSM and CBF increased the variance explained to 37.9% at step 3 (Adjusted *R*^2^ change = 0.043) and 40.6% (Adjusted *R*^2^ change = 0.027) at step 4, respectively.

**Table 3:** Total explained variance (Adjusted *R*^2^) and explained variance attributed to the newest added predictor (Adjusted *R*^2^ change) at each step of the linear regression predicting composite WMH volume.

**Regression Step and Predictors**	**Adjusted *R*^**2**^**	**Adjusted *R*^**2**^ Change**	***p*-value**
Step 1	0.200	0.200	<0.001^*^
Age	–	–	0.001^*^
Sex	–	–	0.417
ICV	–	–	0.025^*^
Step 2	0.336	0.136	<0.001^*^
RD	–	–	<0.001^*^
Step 3	0.379	0.043	<0.001^*^
QSM	–	–	0.018^*^
Step 4	0.406	0.027	<0.001^*^
CBF	–	–	0.046^*^

*Covariates alone predicted 20% of the variance. Including RD at step 2 increased the adjusted R^2^ of the model to 0.336. After inclusion of QSM in step 3 and CBF in step 4, the total variance explained in total WMH volume exceeded 40%. The p-values in bold reflect the p-value of the model at the indicated step, whereas other p-values represent the indicated variable when they were first entered into the model*.

* *p < 0.05*.

### RD, CBF, and QSM Associations With Regional WMH Volumes

Separate linear regressions were next used as a *post-hoc* analysis for the initial ANCOVA to further explore the QSM × WMH volume location interaction, using CBF and RD as covariates. QSM positively predicted deep (bootstrapped Beta = 0.236, partial *r* = 0.307, *F* = 6.841; *p* = 0.034, SE = 0.109, 95% BCa CI = 0.032 to 0.388), but not PV WMH volume (bootstrapped Beta = 0.052, partial *r* = 0.114, *F* = 0.883; *p* = 0.414, SE = 0.063, 95% BCa CI = −0.055 to 0.121; Table 4).

**Table 4:** Summary of main effects and interactions of predictors on WMH volume in periventricular and deep regions.

**WMH Location**	**Periventricular**			**Deep**		
**Predictors**	**Beta**	***F*-value**	***p*-value**	**Beta**	***F*-value**	***p*-value**
Age	0.016	3.453	0.065	0.008	0.341	0.529
Sex	−0.278	6.657	0.009^*^	−0.008	0.002	0.966
ICV	0.126	5.867	0.010^*^	0.118	1.848	0.170
CBF	−0.132	8.387	0.011^*^	−0.074	0.902	0.344
QSM	0.052	0.883	0.414	0.236	6.841	0.034^*^
RD	0.214	16.486	<0.001^*^	0.217	6.908	0.023^*^
CBF × QSM	−0.047	0.814	0.482	−0.054	0.312	0.562
QSM × RD	−0.028	0.125	0.755	0.062	0.339	0.593
CBF × RD	0.013	0.105	0.744	0.012	0.024	0.880

*Displayed are unstandardized beta, F-and p-values for the main effects of all predictors, and interaction effects in both PV (left) and deep (right) WMH models. QSM was positively associated with deep but not PV WMH volume, while RD strongly predicted both. CBF negatively predicted PV WMH volume. There were no interactions between predictors in either model*.

* *p < 0.05*.

### Age Moderation Analysis

Linear regression models were used to explore the possibility of age × CBF, age × QSM, and age × DR interactions. Results indicated that the interaction terms were not statistically significant when predicting PV or deep WMH volume [age × CBF for PV (*p* = 0.986) and for deep (*p* = 0.628); age × QSM for PV (*p* = 0.140) and for deep (*p* = 0.328); and age × DR for PV (*p* = 0.270) and for deep (*p* = 0.778); Supplementary Table 1].

### Power Analysis

If we assume that an interaction effect was present, this study had over 99% power to detect an interaction with a large effect (f^2^ = 0.35), 92% power to detect an interaction with a medium effect (f^2^ = 0.15), and 23% power to detect an interaction with a small effect (f^2^ = 0.15). The actual f^2^ for our most relevant interaction term (QSM × RD) was very small (0.005). This may suggest that the interactions in our models are not truly present; alternatively, the effect may be so small that it would require >1,500 participants to achieve 80% power.

## Discussion

We explored the main effects and interactions of DTI-based radial diffusivity (RD), QSM-based brain iron concentration and ASL-based cerebral blood flow (CBF) on WMH volume and location. Results indicated that RD was the strongest predictor of total WMH volume and was associated with WMH regardless of location. In contrast, iron concentration was more strongly associated with deep than periventricular (PV) WMH volume. Finally, CBF did not account for any unique variance in WMH volume or location after controlling for other predictors. Our findings demonstrate the importance of considering multiple predictors of WMHs simultaneously to identify the strongest individual predictors, after controlling for shared variance between predictors.

### Additive Effects of Individual Predictors of WMH Volume

The covariates and predictors (WM microstructure, brain iron and CBF) explored in this study accounted for almost half of the total variance in composite WMH volume (Table 3). WM microstructure (assessed with RD) contributed the largest percentage of unique variance accounted for in composite WMH volume after accounting for covariates and other predictors, suggesting that WM microstructure is the most robust predictor of the three. While QSM and CBF were also significant predictors, each explained only modest percentages of WMH variance after accounting for covariates and other predictors indicating that a portion of their variance may be better explained by WM microstructure.

### RD Predicts Both PV and Deep WMH Volume

DTI-based RD was strongly associated with WMH volume in both PV and deep locations. Our findings are in-keeping with results from previous cross-sectional studies suggesting that RD (Pelletier et al., 2016) and other DTI-based metrics (Maillard et al., 2011; Maniega et al., 2015; Promjunyakul et al., 2016) appear to predict global WMH volume in both PV and deep WM. However, we know of only one previous study using RD as a WMH predictor that controlled for other neuroimaging predictors (Promjunyakul et al., 2018). In that study, RD values predicted new PV and deep WMH growth ~1.5 years later (Promjunyakul et al., 2018), and was a stronger WMH predictor than CBF.

The present results add to the literature, indicating that RD is associated with WMH volume in both PV and deep regions after controlling for CBF and brain iron concentration. Higher RD is associated with relatively lower myelination, axonal packing and axonal density (Madden et al., 2009). Thus, lower myelination and axonal packing/density appear to be associated with higher WMH volume, both independently of other neuroimaging metrics and across WM location.

### Iron Concentration Is More Closely Associated With Deep Than PV WMH Volume

Non-heme brain iron concentration was quantified using QSM, which has been validated against postmortem tissue iron concentrations in both subcortical structures (Langkammer et al., 2012; Sun et al., 2015) and cortical structures (Fukunaga et al., 2010; Bulk et al., 2018). Our QSM ROI included both cortical and subcortical structures. We observed a significant main effect of cerebral iron concentration on total WMH volume after controlling for WM microstructural properties and cerebral blood flow. While non-heme iron is crucial for many cellular processes, it is also is a potent oxidizer that can generate reactive oxygen species (ROS), damaging neurons (Moos et al., 2007; Ward et al., 2014). Increased unbound iron is thought to contribute to demyelination resulting from free radical damage affecting oligodendrocytes and myelin sheaths (Todorich et al., 2009; Bartzokis, 2011). However, given that we controlled for myelin damage in our models (DTI-based RD was used as a covariate), the main effect of QSM on WMH we observed could reflect additional damage to neuronal cell bodies and/or axons (e.g., Wallerian degeneration).

One reason why iron was less predictive of total WMH volume than RD relates to the interaction we observed between brain iron and location on WMH volume. Specifically, brain iron concentration was more predictive of deep WMH volume than PV WMH volume. Several previous studies have had mixed findings, with some indicating that iron is associated with total WMH volume (Yan et al., 2013; Valdés Hernández et al., 2016; Sun et al., 2017), although others have not (Gattringer et al., 2016). However, none of these studies accounted for CBF or FA/RD in their models or explored potential iron by WMH location interactions.

The interaction we observed between iron and location on WMH volume likely relates to deep WM ROIs containing more tracts with connections to iron-rich basal ganglia and other subcortical structures in our study. In particular, the deep ROI included the external capsule, a series of WM tracts situated between the putamen and claustrum. The external capsule includes extensive connections between the claustrum and subcortical structures such as the basal ganglia and thalamus (Smythies et al., 2013). In contrast, the PV WM ROI was primarily composed of the body and splenium of the corpus callosum, which connect homologous neocortical structures.

It is well-established that age-related cerebral iron accumulation is seen predominantly in subcortical structures of the basal ganglia (Zecca et al., 2004; Ward et al., 2014). Thus, it is possible that high concentrations of iron in basal ganglia structures may have contributed to Wallerian degeneration of deep tracts such as the external capsule. However, it should be noted that our analyses focused on major WM tracts. There are a number of smaller WM tracts in the PV region with connections to basal ganglia structures such as the lenticular fasciculus and nigrostriatal tracts which were not included in our study due to less optimal spatial alignment across participants.

### CBF Does Not Predict WMH Volume After Controlling for Other Factors

Our results showed that CBF was negatively associated with total WMH volume when it was the only predictor of interest. This is consistent with results from a number of studies reporting a negative relationship between CBF and WMH volume (ten Dam et al., 2007; Brickman et al., 2009; Bahrani et al., 2017). However, our results indicated that CBF was only a marginally significant predictor of total WMH volume after controlling for other predictors (*p* = 0.065). In particular, we found that RD accounted for some of the variance in the CBF-WMH volume relationship (Section CBF Association With WMH Volume in Partial Models), and better predicted WMH volume than CBF in general (Table 2, section Additive Effects of WMH Predictors Using *R*^2^ Change). One possible explanation for this is that CBF only predicts total WMH volume when reduced blood flow is pronounced enough to cause microstructural damage to myelin and WM tracts.

### Age Did Not Moderate Relationships Between Predictors (CBF, QSM, DR) and WMH Volume

It is possible that CBF, QSM, or DR could better predict WMH volume at older age levels, as all these measures are known to correlate with age (Acosta-Cabronero et al., 2016; Beck et al., 2021; Juttukonda et al., 2021). We investigated this possibility by exploring whether age moderated the strength of relationship between any of our predictors and WMH volume. However, we did not find any evidence of age moderation in this study (Supplementary Table 1). As our study included mostly healthy older adults, future studies could examine the possibility that age may moderate the relationship between cSVD predictors and WMH volume at more advanced neurodegenerative states, such as those associated with mild cognitive impairment or dementia.

### Study Strengths and Limitations

Strengths of our study include the consideration of multiple predictors (WM microstructure, brain iron concentration and CBF) of WMH volume, employing rigorous MRI analyses, a moderately large sample size and wide age range of older adults. Further, the integration of multiple WMH predictors in the same model controls for shared covariance between predictors, allowing identification of the best predictors through their unique variance on WMH volume. An additional strength of our study was consideration of interactions between predictors and spatial location (i.e., PV and deep WMHs).

This study also has limitations that highlight the need for additional follow-up studies. First, our cross-sectional study cannot determine how predictors would be associated with WMH growth. Future research with multiple time points is needed to identify baseline predictors of longitudinal WMH change. Second, as is true in most studies, ASL in our study had poorer spatial resolution than either DTI or QSM. The mean CBF signal used in our study consisted of a number of individual gray matter ROIs, including subcortical structures such as the caudate and putamen. Subcortical structures are surrounded by white matter where ASL signal is reduced compared to gray matter, and relatively large ASL voxels may have contributed to greater partial volume effects and averaging of lower CBF signal. For example, the putamen is bordered laterally by the external capsule, a long, thin WM tract. FreeSurfer segmentation, which we used, tends to overestimate the boundaries of the putamen by including the external capsule (Dewey et al., 2010; Perlaki et al., 2017). Although CBF did not predict total WMH volume when other predictors were controlled in our study, it is possible that CBF could contribute unique variance in total WMH volume using more advanced ASL techniques that are currently in development. Third, future studies should explore more than one clinical subset of participants. Our study focused on cognitively normal older adults and it is possible that our results may not generalize to individuals with more advanced WMH burden and cognitive impairment (Mild Cognitive Impairment and Alzheimer's Disease). Longitudinal follow-up is needed to identify if synergistic interactions begin to emerge at the onset of dementia.

This study employed a large-scale ROI approach to assess effects of more global CBF/QSM/DR predictors on overall measures of WMH volume (including total, PV and deep regions). In previous work in this area, both large-scale ROI approaches (ten Dam et al., 2007; Brickman et al., 2009; Leritz et al., 2014; Pelletier et al., 2016; Wiseman et al., 2018) and voxelwise or small ROI approaches (Maillard et al., 2011, 2014; Promjunyakul et al., 2016, 2018) have been employed. Each of these approaches have strengths and limitations. For example, local/small ROI approaches have the potential to reveal fine-grained anatomical relationships but may have limitations associated with imperfect registration between imaging modalities. In contrast, large-scale ROI approaches such as the one used here may “blur” some fine-grained anatomical associations, but have the advantage of identifying network-level associations. Finally, as with several previous studies (ten Dam et al., 2007; Griffanti et al., 2018), this study focused on deep vs. PV WMH regions. Future work should explore the possibility that WMH predictors may have differential effects in additional ROIs, such as in lobar ROIs.

## Conclusion

WM microstructure is strongly associated with both total and regional WMH volumes, even after controlling for other predictors. Brain iron concentration is also a significant predictor but adds only modest unique variance due to being selectively associated with deep but not PV WMH volume. Future studies should attempt to further clarify which predictors contribute additional, unique variance in WMH volume after controlling for WM microstructure and brain iron concentration.

## Data Availability Statement

The raw data supporting the conclusions of this article will be made available by the authors, without undue reservation.

## Ethics Statement

The studies involving human participants were reviewed and approved by Institutional Review Board of the University of Kentucky. The patients/participants provided their written informed consent to participate in this study.

## Author Contributions

CB: conceptualization, data collection, data curation, methodology, formal analysis, writing (original draft), and writing (review and editing). VZ: data collection, data curation, methodology, formal analysis, visualization, and writing (review and editing). ES: data collection, data curation, methodology, and investigation. BG: conceptualization, data curation, methodology, formal analysis, writing (review and editing), supervision, project administration, and funding acquisition. All authors contributed to the article and approved the submitted version.

## Conflict of Interest

The authors declare that the research was conducted in the absence of any commercial or financial relationships that could be construed as a potential conflict of interest.

## Abbreviations

cSVD: Cerebral Small Vessel Disease
MRI: Magnetic Resonance Imaging
WMH: White Matter Hyperintensity
PV: Periventricular
DTI: Diffusion Tensor Imaging
FA: Fractional Anisotropy
RD: Radial Diffusivity
CBF: Cerebral Blood Flow
QSM: Quantitative Susceptibility Mapping
GM: Gray Matter.
