# Supplementary material for: White Matter Hyperintensity Volume and Location: Associations With WM Microstructure, Brain Iron, and Cerebral Perfusion
Source: Front Aging Neurosci. 2021 Jul 5;13:617947. doi: 10.3389/fnagi.2021.617947 (PMC8287527; doi:10.3389/fnagi.2021.617947)
Supplement: Supplementary file 1 [file Table_1.docx]

**Table S1** Summary of main effects and age × predictor interactions on WMH volume in periventricular and deep regions.

| Table S1 |  |  |
| --- | --- | --- |
| WMH Location | Periventricular | Deep |
| Predictors | *p*-value | *p*-value |
| Age | 0.013* | 0.211 |
| Sex | 0.008* | 0.603 |
| ICV | 0.066 | 0.349 |
| CBF | 0.018* | 0.161 |
| QSM | 0.424 | 0.021* |
| RD | <0.001* | 0.024* |
| Age × CBF | 0.986 | 0.628 |
| Age × QSM | 0.140 | 0.328 |
| Age × DR | 0.270 | 0.778 |

*Displayed are the p-values for the main effects of all predictors, and interaction terms in both PV (left) and deep (right) WMH models. There were no statistically significant interaction terms in either model.*

**p < 0.05*
